# Structural basis of glycogen branching enzyme deficiency and pharmacologic rescue by rational peptide design

**DOI:** 10.1093/hmg/ddv280

**Published:** 2015-07-21

**Authors:** D. Sean Froese, Amit Michaeli, Thomas J. McCorvie, Tobias Krojer, Meitav Sasi, Esther Melaev, Amiram Goldblum, Maria Zatsepin, Alexander Lossos, Rafael Álvarez, Pablo V. Escribá, Berge A. Minassian, Frank von Delft, Or Kakhlon, Wyatt W. Yue

**Affiliations:** 1 Structural Genomics Consortium, Nuffield Department of Clinical Medicine, University of Oxford, OX3 7DQ, UK,; 2 Pepticom LTD, Jerusalem, Israel,; 3 Department of Neurology, Hadassah-Hebrew University Medical Center, Ein Kerem, Jerusalem, Israel,; 4 Institute for Drug Research, The Hebrew University of Jerusalem, Jerusalem, Israel,; 5 Department of Biology, University of the Balearic Islands, Palma de MallorcaE-07122, Spain and; 6 Program in Genetics and Genomic Medicine, The Hospital for Sick Children, University of Toronto, Toronto, Canada

## Abstract

Glycogen branching enzyme 1 (GBE1) plays an essential role in glycogen biosynthesis by generating α-1,6-glucosidic branches from α-1,4-linked glucose chains, to increase solubility of the glycogen polymer. Mutations in the *GBE1* gene lead to the heterogeneous early-onset *glycogen storage disorder type IV* (GSDIV) or the late-onset *adult polyglucosan body disease* (APBD). To better understand this essential enzyme, we crystallized human GBE1 in the apo form, and in complex with a tetra- or hepta-saccharide. The GBE1 structure reveals a conserved amylase core that houses the active centre for the branching reaction and harbours almost all GSDIV and APBD mutations. A non-catalytic binding cleft, proximal to the site of the common APBD mutation p.Y329S, was found to bind the tetra- and hepta-saccharides and may represent a higher-affinity site employed to anchor the complex glycogen substrate for the branching reaction. Expression of recombinant GBE1-p.Y329S resulted in drastically reduced protein yield and solubility compared with wild type, suggesting this disease allele causes protein misfolding and may be amenable to small molecule stabilization. To explore this, we generated a structural model of GBE1-p.Y329S and designed peptides *ab initio* to stabilize the mutation. As proof-of-principle, we evaluated treatment of one tetra-peptide, Leu-Thr-Lys-Glu, in APBD patient cells. We demonstrate intracellular transport of this peptide, its binding and stabilization of GBE1-p.Y329S, and 2-fold increased mutant enzymatic activity compared with untreated patient cells. Together, our data provide the rationale and starting point for the screening of small molecule chaperones, which could become novel therapies for this disease.

## Introduction

Glycogen is a compact polymer of α-1,4-linked glucose units regularly branched with α-1,6-glucosidic bonds, serving as the main carbohydrate store and energy reserve across many phyla (1). In eukaryotes, *glycogenin* (EC 2.4.1.186) initiates the synthesis of the linear glucan chain (2), which is elongated by *glycogen synthase* (GYS, EC 2.4.1.11) (3), functioning in concert with *glycogen branching enzyme* (GBE, EC 2.4.1.18) to introduce side chains (4). GBE (also known as 1,4-glucan:1,4-glucan 6-glucanotransferase) transfers α-1,4-linked glucose units from the outer ‘non-reducing’ end of a growing glycogen chain into an α-1,6 position of the same or neighbouring chain, thereby creating glycogen branches. Together GYS and GBE define the globular and branched structure of glycogen, which increases its solubility by creating a hydrophilic surface (5) and regulates its synthesis by increasing the number of reactive termini for GYS-mediated chain elongation (6). Similar branching enzyme activities are also found in plants, using amylopectin as substrate (7).

Inherited mutations in the human *GBE1* (hGBE1) gene (chromosome 3p12.3) (5) cause the autosomal recessive glycogen storage disorder type IV (GSDIV; OMIM 232500) (8,9). GSDIV constitutes ∼3% of all GSD cases (10) and is characterized by the deposition of an amylopectin-like polysaccharide that has fewer branch points, longer outer chains and poorer solubility than normal glycogen. This malconstructed glycogen (termed *polyglucosan*), presumably the result of GYS activity outpacing that of mutant GBE, accumulates in most organs including liver, muscle, heart and the central and peripheral nervous systems, leading to tissue and organ damage, and cell death. GSDIV is an extremely heterogeneous disorder with variable onset age and clinical severity, including a classical hepatic form in neonates and children that progresses to cirrhosis (Andersen disease) (11), a neuromuscular form classified into four subtypes (perinatal, congenital, juvenile, adult onset) (12), as well as a late-onset allele variant—adult polyglucosan body disease (APBD, OMIM 263570)—a neurological disorder affecting mainly the Ashkenazi Jewish population (13). To date there is no transformative treatment for GSDIV and APBD. A majority of disease-causing mutations are of the missense type, likely to affect the GBE protein structure and function (14).

GBE is classified as a carbohydrate-active enzyme (http://www.cazy.org) and catalyses two reactions presumably within a single active site. In the first reaction (*amylase-type hydrolysis*), GBE cleaves every 8–14 glucose residues of a glucan chain, an α-1,4-linked segment of more than six glucose units from the non-reducing end. In the second reaction (*transglucosylation*), it transfers the cleaved oligosaccharide (‘donor’), via an α-1,6-glucosidic linkage, to the C6 hydroxyl group of a glucose unit (‘acceptor’) within the same chain (*intra-*) or onto a different neighbouring chain (*inter-*). The mechanistic determinants of the branching reaction, e.g. length of donor chain, length of transferred chain, distance between two branch points, relative occurrence of intra- versus inter-chain transfer and variation among organisms, remain poorly understood.

Almost all sequence-annotated branching enzymes, including those from diverse organisms, belong to the GH13 family of glycosyl hydrolases (also known as the α-amylase family) (5) and fall either into subfamily 8 (eukaryotic GBEs) or subfamily 9 (prokaryotic GBEs) (15). The GH13 family is the largest glysoyl hydrolase family, comprised of amylolytic enzymes (e.g. amylase, pullulanase, cyclo-maltodextrinase and cyclodextrin glycosyltransferase) that carry out a broad range of reactions on α-glycosidic bonds, including hydrolysis, transglycosylation, cyclization and coupling. These enzymes share a (β/α)_8_ barrel domain with an absolutely conserved catalytic triad (Asp-Glu-Asp) at the C-terminal face of the barrel (16). In several GH13 enzymes, this constellation of three acidic residues functions as the nucleophile (Asp357, hGBE1 numbering hereinafter), proton donor (Glu412) and transition state stabilizer (Asp481) in the active site. To date, crystal structures available from GH13-type GBEs from plant (17) and bacteria (18,19) have revealed an overall conserved architecture; however, no mammalian enzyme has yet been crystallized. In this study, we determined the crystal structure of hGBE1 in complex with oligosaccharides, investigated the structural and molecular bases of disease-linked missense mutations and provided proof-of-principle rescue of mutant hGBE1 activity by rational peptide design.

## Results and Discussion

### hGBE1 structure determination

For structural studies, we pursued baculovirus-infected insect cell overexpression of hGBE1, a 702-amino acid (aa) protein. Interrogation of several N- and C-terminal boundaries (Supplementary Material, Fig. S1) in this expression system yielded a soluble and crystallisable polypeptide for hGBE1 from aa 38–700 (hGBE1_trunc_). Using the molecular replacement method with the *Oryza sativa* starch branching enzyme I (SBE1; PDB: 3AMK; 54% identity to hGBE1) as search model, we have determined the structure of hGBE1_trunc_ in the *apo* form (hGBE1-apo), and in complex with the tetra-saccharide acarbose (hGBE1-ACR) or hepta-saccharide maltoheptaose (hGBE1-Glc7), to the resolution range of 2.7–2.8 Å (Supplementary Material, Table S1). Inspection of the asymmetric unit content as well as symmetry-related protomers did not reveal any stable oligomer arrangements, consistent with GBE1 being a monomer in size-exclusion chromatography (data not shown), similar to most GH13 enzymes.

**Figure 1.**
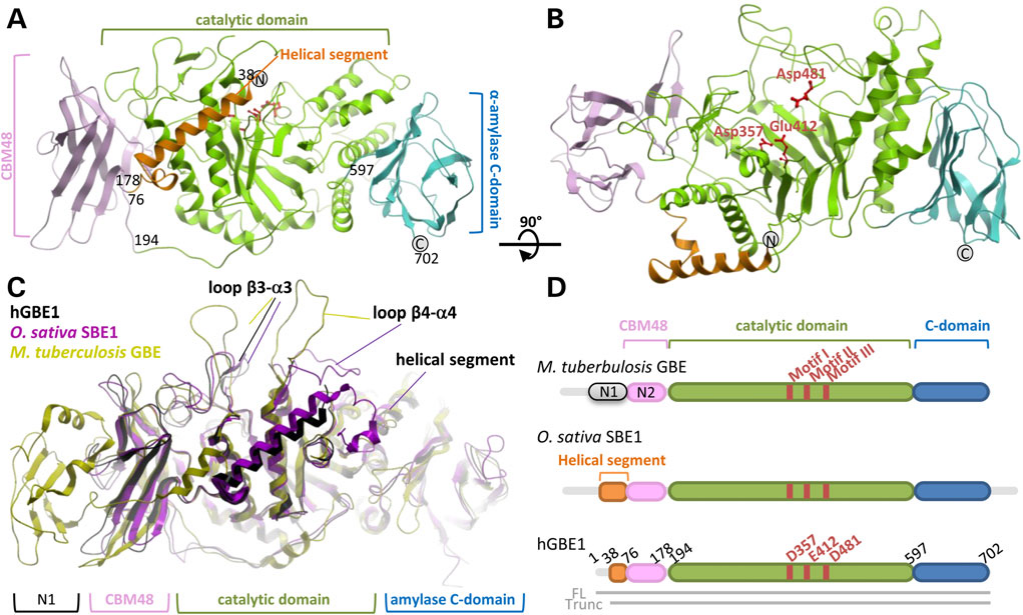
Crystal structure of hGBE1. (**A** and **B**) Orthogonal views of hGBE1 showing the N-terminal helical segment (orange), CBM48 (pink), central catalytic domain (green) and C-terminal domain (blue). The catalytic triad Asp357-Glu412-Asp481 is shown as red sticks. Numbers refer to domain boundaries. N- and C-termini are labelled as grey spheres. (**C**) Superposition of branching enzyme structures from human (hGBE1, this study), *O. sativa* SBE1 and *M. tuberculosis* GBE, highlighting the conserved domain architecture and three regions of structural variation. (**D**) Domain organization of hGBE1, *O. sativa* SBE1 and *M. tuberculosis* GBE revealing differences in the N-terminus between prokaryotic and eukaryotic polypeptides. Prokaryotic GBEs contain two N-terminal carbohydrate-binding domains (N1, N2) whereas eukaryotes contain only one (CBM48) and replace the prokaryotic N1 domain with a helical extension.

hGBE1 is an elongated molecule (longest dimension >85 Å) composed of four structural regions (Fig. 1A and B): the N-terminal helical segment (aa 43–75), a carbohydrate-binding module 48 (CBM48; aa 76–183), a central catalytic core (aa 184–600) and the C-terminal amylase-like barrel domain (aa 601–702). A structural overlay of hGBE1 with reported branching enzyme structures from *O. sativa* SBE1 (17) (PDB: 3AMK, C^α^-RMSD: 1.4 Å, sequence identity: 54%) and *M. tuberculosis* GBE (19) (3K1D, 2.1 Å, 28%) (Fig. 1C) highlights the conserved catalytic core housing the active site within a canonical (βα)_6_ barrel (16). Nevertheless, the different branching enzymes show greater structural variability in the N-terminal region preceding the catalytic core, as well as in two surface-exposed loops of the TIM barrel (Fig. 1C). For example, in *O. sativa* SBE1 and human GBE1 structures, the helical segment precedes the CBM48 module, whereas in *M. tuberculosis* GBE, the helical segment is replaced by an additional β-sandwich module (N1 in Fig. 1C and D). The closer homology of hGBE1 with *O. sativa* SBE1, whose substrate is starch, than with the bacterial paralog *M. tuberculosis* GBE, suggests a similar evolutionary conservation in the branching enzyme mechanism for glycogen and starch, both involving a growing linear α1,4-linked glucan chain as substrate.

### Oligosaccharide binding of hGBE1 at catalytic and non-catalytic sites

**Figure 2.**
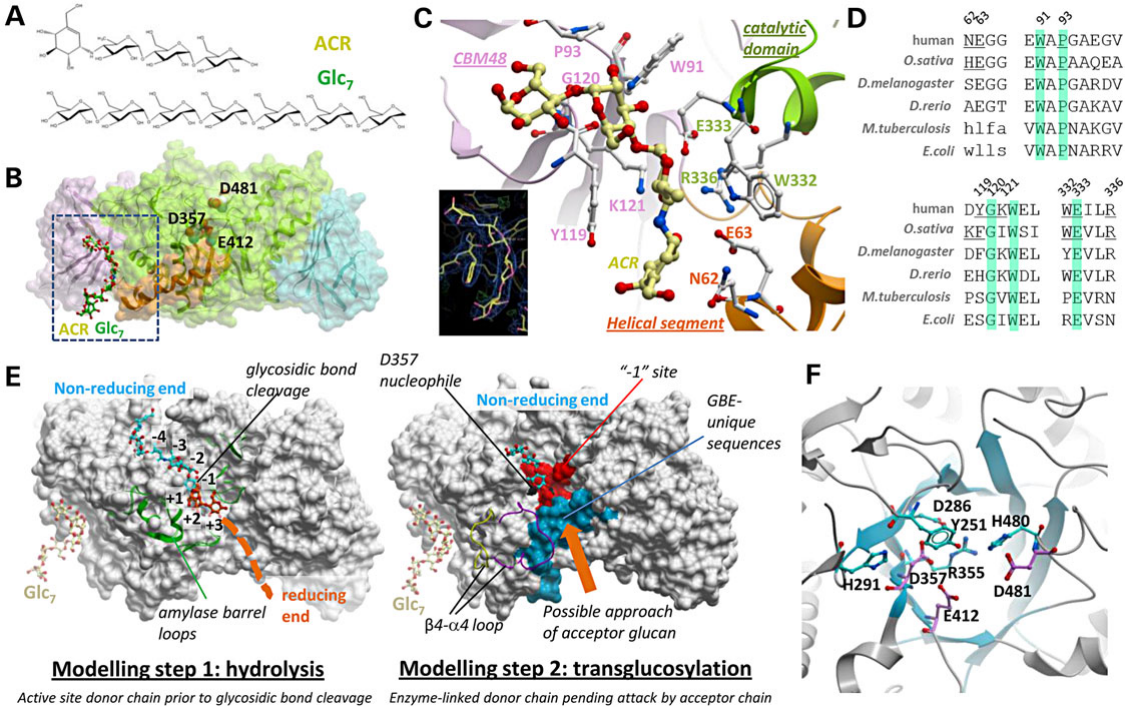
Oligosaccharide binding to hGBE1. (**A**) Chemical structures of acarbose (ACR) and (Glc_7_). (**B**) Surface representation of hGBE1 (Fig. 1A colouring) showing the bound oligosaccharides. (**C**) ACR binding cleft at the interface of the helical segment (orange), CBM48 (pink) and catalytic domain (green). Shown in sticks are ACR (yellow carbon atoms) and its contact protein residues (white carbon atoms). Inset, 2Fo-Fc electron density for the modelled ACR. (**D**) Sequence alignment of the ACR-binding residues of hGBE1 (underlined). Annotated branching enzyme sequences are from human (Uniprot ID Q04446), *O. sativa* SBE1 (Q01401), *D. melanogaster* (A1Z992), *D. rerio* (F8W5I0), *M. tuberculosis* (P9WN45) and *E. coli* (P07762). (**E**) Surface representation of the hGBE1–Glc_7_ complex to model the two GBE reaction steps. Left panel is overlayed with a decasaccharide ligand (blue and orange stick) and TIM barrel loops (green ribbon) from the *B. amyloliquefaciens* and *B. licheniformis* chimeric amylase structure (PDB code 1e3z) to highlight the broader active site cleft in hGBE1 owing to the absence of these amylase loops. Right panel is overlayed with maltotriose (cyan stick) from pig pancreatic α-amylase (PDB code 1ua3), as well as the β4-α4 loop from *O. sativa* SBE1 (purple) and *M. tuberculosis* GBE (yellow) structures, which is disordered in hGBE1. Superposition of hGBE1 with structural homologs is illustrated in Supplementary Material, Figure S3. (**F**) Close-up view of the hGBE1 active site barrel (cyan strands) that harbours the conserved residues (sticks) of the ‘*−*1’ subsite. Residues constituting the putative catalytic triad are coloured magenta.

To characterize the binding of oligosaccharides to branching enzymes, we co-crystallized hGBE1_trunc_ with acarbose (ACR) or maltoheptaose (Glc7) (Fig. 2A). ACR is a pseudo-tetra-saccharide acting as active site inhibitor for certain GH13 amylases. In the hGBE1-ACR structure, acarbose is bound not at the expected active site but instead at the interface between the CBM48 and the catalytic domains (Fig. 2B). Within this oligosaccharide binding cleft (Fig. 2C), ACR interacts with protein residues from the N-terminal helical segment (Asn62 and Glu63), CBM48 domain (Trp91, Pro93, Tyr119, Gly120 and Lys121) as well as catalytic core (Trp332, Glu333 and Arg336). These interactions, likely to be conserved among species (Fig. 2D), include hydrogen bonds to the sugar hydroxyl groups as well as hydrophobic/aromatic interactions with the pyranose rings. The hGBE1-Glc7 structure reveals similar conformation and binding interactions of maltoheptaose for its first four 1,4-linked glucose units (Fig. 2B). The three following glucose units, however, extend away from the protomer surface and engage in interactions with a neighbouring non-crystallographic symmetry (NCS)-related protomer in the asymmetric unit (Supplementary Material, Fig. S2A). These artifactual interactions mediated by crystal packing are unlikely to be physiologically relevant.

CBM48 is a β-sandwich module found in several GH13 amylolytic enzymes (20). The acarbose binding cleft observed here is the same location that binds maltopentaose in the *O. sativa* SBE1 structure (21), as well as other oligosaccharides in CBM48-containing proteins (Supplementary Material, Fig. S2B). The conserved nature of this non-catalytic cleft among branching enzymes (Fig. 2D), and its presumed higher affinity for oligosaccharides than the active site, may represent one of the multiple non-catalytic binding sites on the enzyme surface. They may provide GBEs the capability to anchor a complex glycogen granule and, as proposed previously (22), determine the chain length specificity for the branching reaction as a ‘molecular ruler’. This agrees with the emerging concept of glycogen serving not only as the substrate and product of its metabolism but also as a scaffold for all acting enzymes.

In light of our unsuccessful attempts to co-crystallize hGBE1 with an active site-bound oligosaccharide, our analysis of the active site is guided by reported structures of GH13 α-amylases in complex with various oligosaccharides (23,24) (Supplementary Material, Fig. S3A). The catalytic domain TIM barrel of hGBE1 superimposes well with those from the amylase structures (RMSD 1.2 Å for 130–150 C^α^ atoms; Supplementary Material, Fig. S3B), suggesting a similar mode of substrate threading along the GH13 enzyme active sites, at least within the proximity of glycosidic bond cleavage. The hGBE1 active site is a prominent surface groove at the (βα)_6_-barrel that could bind a linear glucan chain via a number of subsites (Fig. 2E, left), each binding a single glucose unit. The subsites are named ‘*–n*’, … ‘−1’, ‘+1’, … ‘+*n’*, denoting the *n-*th glucose unit in both directions from the scissile glycosidic bond. The most conserved among GH13 enzymes is the ‘*−*1’ subsite, which harbours seven strictly conserved residues forming the catalytic machinery (16) (Fig. 2F and Supplementary Material, Fig. S4A). The other subsites lack a significant degree of sequence conservation, suggesting that substrate recognition other than at the ‘*−*1’ subsite is mediated by surface topology and shape complementarity, and not sequence-specific interactions.

The hGBE1 active site is tasked to catalyse two reaction steps (hydrolysis and transglucosylation) on a growing glucan chain (Supplementary Material, Fig. S5). The first reaction is a nucleophilic attack on the ‘*−*1’ glucose at the C-1 position by an aspartate (Asp357), generating a covalent enzyme–glycosyl intermediate with release of the remainder of the glucan chain carrying the reducing end (+1, +2 …). In the second reaction, the enzyme-linked ‘*−*1’ glucose is attacked by a glucose 6-hydroxyl group from either the same or another glucan chain, which acts as a nucleophile for the chain transfer. While both hGBE1 reactions presumably proceed via a double displacement mechanism involving the strictly conserved triad Asp357-Glu412-Asp481, as proposed for GH13 amylases, there exist mechanistic differences between branching and amylolytic enzymes: (i) the branching enzyme substrate is not a malto-oligosaccharide, but rather a complex glycogen granule with many glucan chains and (ii) the transglycosylation step in GBE (glucose 6-OH as acceptor) is replaced by hydrolysis in amylases (H_2_O as acceptor). These differences require that the active site entrance of hGBE1 be tailor-made to accommodate the larger more complex glucose acceptor chain (Fig. 2E), as opposed to a water molecule in amylases. A region of GBE-unique sequences (aa 405–443), rich in Gly/Ala residues, has been identified based on alignment with GH13 sequences (25) (Supplementary Material, Fig. S4B). This region, replaced in amylolytic enzymes by sequence insertions and bulkier residues, maps onto a hGBE1 surface that is proximal to the ‘+1, +2 …’ subsites, and to the β4-α4 loop that is disordered in hGBE1 but adopts different conformations in the *O. sativa* and *M. tuberculosis* structures (Figs. 1B and 2E, right). We posit that this surface region, unique to branching enzymes, facilitates access to the active site by an incoming glucan acceptor chain. While beyond the scope of this work, it will be of interest to determine how this GBE-unique region and the non-catalytic oligosaccharide binding cleft function together to bind the complex glycogen granule.

### GBE1 missense mutations are predominantly localized in the catalytic core

**Figure 3.**
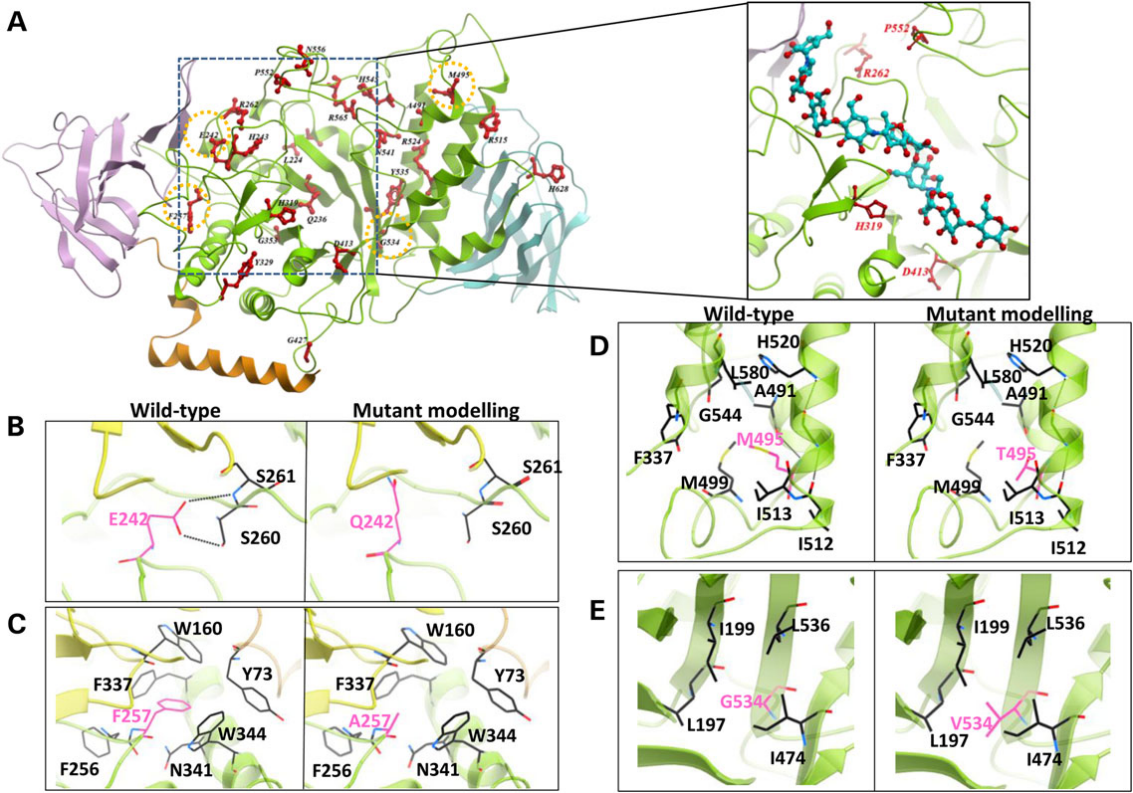
Structural analysis of hGBE1 mutations. (**A**) Mapping of disease-associated missense mutation sites (red sticks) on the hGBE1 structure underlines their prevalence in the central catalytic core. Inset—view of the hGBE1 sites showing four missense mutation sites (red sticks), which could be involved in binding a glucan chain, indicated by an overlayed decasacharide ligand from the 1e3z structure. (**B–D**) Structural environment of representative mutation sites, shown in (A) by orange-dotted circles, to illustrate how a single amino acid substitution (pink sticks) may: abolish hydrogen-bonding interactions (B, p.E242Q), create a water-filled cavity within a hydrophobic core (C, p.F257A), abolish hydrophobic interactions (D, p.M495T) and create steric clash with neighbouring residues (E, p.G534V). The mutant models are generated by simple side-chain replacement from the wild-type coordinates.

The hGBE1 crystal structure provides a molecular framework to understand the pathogenic mutations causing GSDIV and APBD, as the previously determined bacterial GBE structures have low amino acid conservation in some of the mutated positions. Apart from a few large-scale aberrations (nonsense, frameshift, indels, intronic mutations), which likely result in truncated and non-functional enzyme, there are to date 25 reported *GBE1* missense mutations, effecting single amino acid changes at 22 different residues (Supplementary Material, Table S2). These mutation sites are predominantly localized in the catalytic core (Fig. 3A), with a high proportion around exon 12 (*n* = 6 in exon 12, *n* = 2 in exon 13, *n* = 1 in exon 14) (26). There is no apparent correlation among the genotype, amino acid change and its associated disease phenotype. However, inspection of the atomic environment surrounding these residues, some of which are strictly invariant among GBE orthologs (Supplementary Material, Fig. S6), allows us to postulate their molecular effects. They can be classified into ‘destabilising’ substitutions, which likely disrupt protein structure, and ‘catalytic’ substitutions, which are located proximal to the active site and may affect oligosaccharide binding or catalysis. The most common type of ‘destabilising’ mutations is those disrupting H-bond networks (p.Q236H, p.E242Q, p.H243R, p.H319R/Y, p.D413H, p.H545R, p.N556Y, p.H628R; Fig. 3B) and ionic interactions (p.R262C, p.R515C/H, p.R524Q, p.R565Q) within the protein core, whereas disruption of aromatic or hydrophobic interactions are also common (p.F257L, p.Y329S/C, p.Y535C, p.P552L; Fig. 3C). Also within the protein core, mutation of a large buried residue to a small one creates a thermodynamically unfavoured cavity (p.M495T, p.Y329S/C; Fig. 3D), whereas mutation from a small residue to a bulkier one creates steric clashes with the surroundings (p.G353A, A491Y, p.G534V; Fig. 3E). In certain cases, mutation to a proline within an α-helix likely disrupts local secondary structure (e.g. p.L224P), whereas mutation from glycine can lose important backbone flexibility (e.g. p.G427R, likely causing Gln426 from the catalytic domain to clash with Phe45 in the helical segment). The ‘catalytic’ mutations are more difficult to define in the absence of a sugar bound hGBE1 structure at the active site. However, superimposing hGBE1 with amylase structures reveals Arg262, His319, Asp413 and Pro552 as mutation positions that could line the oligosaccharide access to the active site (Fig. 3A, inset). In particular, the imidazole side-chain of His319 is oriented towards the active site and within 8 Å distance from the *−*1 site. Its substitution to a charged (p.H319R) or bulky (p.H319Y) amino acid could potentially destabilize oligosaccharide binding.

### GBE1 p.Y329S is a destabilizing mutation

**Figure 4.**
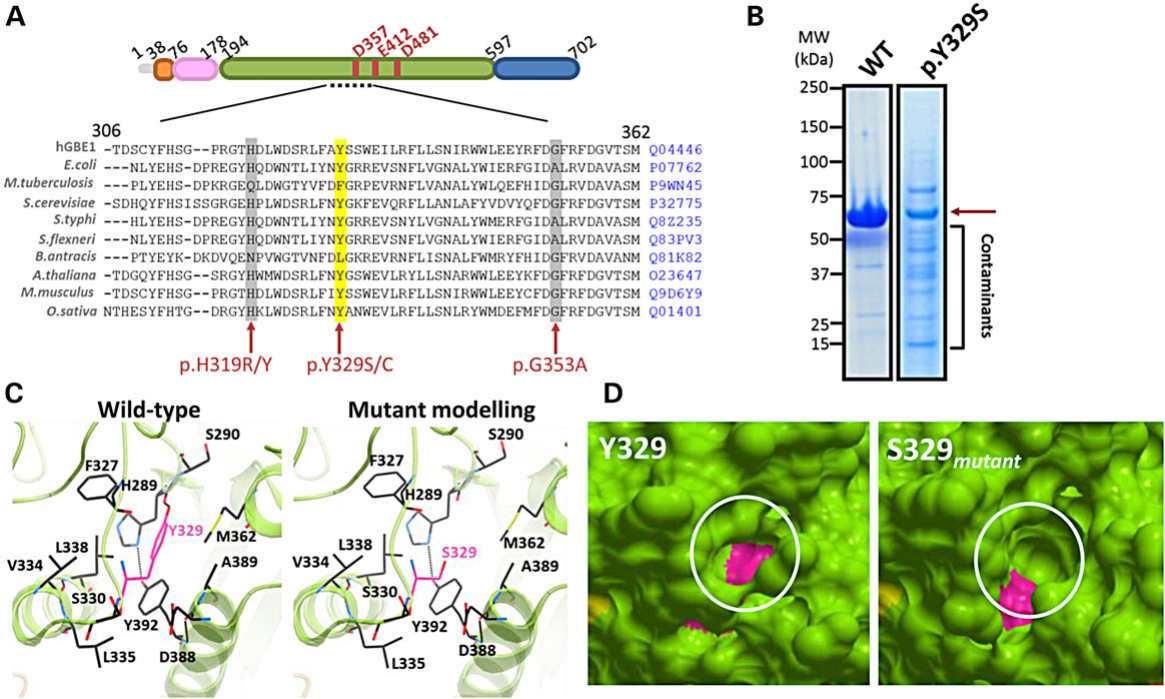
p.Y329S mutation results in destabilized hGBE1 protein. (**A**) Tyr329 is highly conserved across various GBE orthologs (Uniprot ID for each sequence is shown). (**B**) SDS–PAGE of affinity-purified hGBE1 WT and p.Y329S, showing much reduced level of soluble mutant protein. (**C**) Structural analysis of Tyr329 and its neighbourhood reveals a number of hydrophobic interactions that are removed by its substitution with serine. (**D**) Tyr329 (magenta, left panel) is accessible to the protein exterior, and its mutation to Ser329 (magenta, right panel) creates a cavity (circled).

The c.986A>C mutation results in the p.Y329S amino acid substitution, the most common APBD-associated mutation (27). This residue is highly conserved across different GBE orthologs supporting its associated pathogenicity (Fig. 4A). We observed drastically reduced recombinant expression and protein solubility from an hGBE1 construct harbouring the p.Y329S substitution, compared with wild type (Fig. 4B). We therefore sought a molecular explanation by inspecting our hGBE1 structure. Tyr329 is a surface-exposed residue in the catalytic domain and confers stability to the local environment by interacting with the hydrophobic residues Phe327, Val334, Leu338, Met362 and Ala389. Additionally, the tyrosyl hydroxyl group hydrogen bonds with the His289 backbone oxygen (Fig. 4C, left). Mutation of Tyr329 to the smaller amino acid serine (Ser329_mutant_) likely removes these interactions (Fig. 4C, right) and creates a solvent accessible cavity within this hydrophobic core (Fig. 4D), both of which could lead to destabilized protein. Together, our expression and structural analyses demonstrate that the p.Y329S mutation commonly associated with APBD results in protein destabilization.

### Computational design of hGBE1 p.Y329S-stabilizing peptide

We next investigated whether the p.Y329S-associated protein destabilization could be ‘rescued’ by pharmacological chaperone treatment (28). To facilitate the design of a small molecule/peptide chaperone, which could confer stability to the Ser329_mutant_ site, we first generated a structural model of hGBE1-Y329S from the wild-type hGBE1-apo coordinates. Using the assumption that the hGBE1-apo crystal structure represents an active enzyme conformation, the design of an hGBE1 p.Y329S-stabilizing peptide was performed using a rigid backbone modelling of the mutation, in order to retain maximum similarity to the active enzyme.

**Figure 5.**
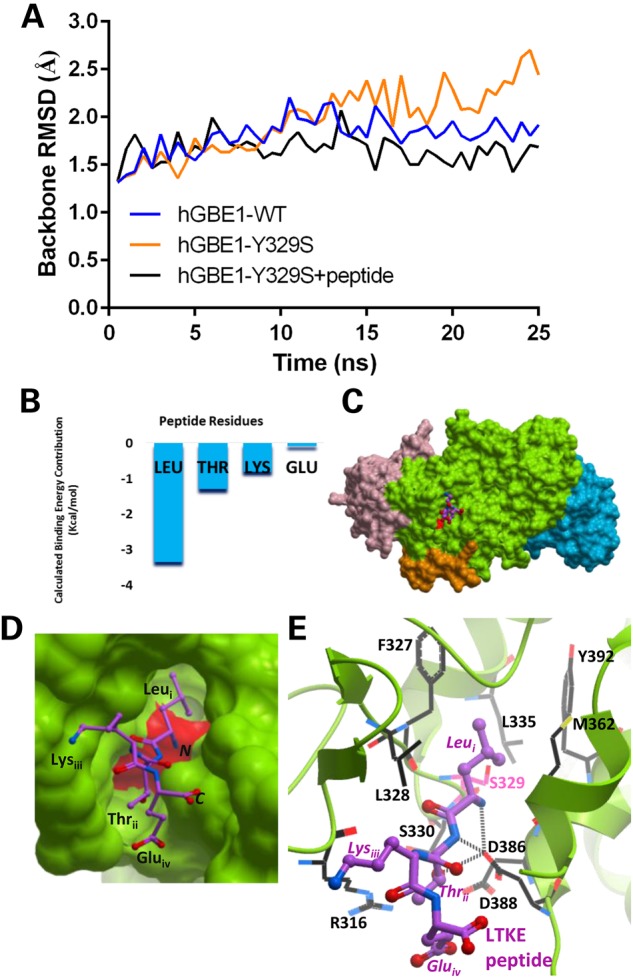
*In silico* peptide design to fill the Ser239_mutant_ cavity. (**A**) Root mean-squared deviation (RMSD) from the backbone as a representation of structural stability (29). The dynamic conformations of the three structural models (hGBE1 WT, hGBE1-Y329S and peptide-bound hGBE1-Y329S) at 0.5-ns intervals along the simulation were superimposed with the initial backbone of the WT structure, in order to obtain RMSD values. (**B**) The molecular mechanics force field calculated binding free energy contributions of individual amino acids in the tetra-peptide LTKE. The Leu N-terminus contributes more than half of total binding free energy. (**C**) Homology model of hGBE1-Y329S in complex with the LTKE peptide (purple sticks) at the Ser329_mutant_ cavity. (**D**) Close-up view of the LTKE peptide, where the side-chain of the N-terminal leucine (Leu_i_) residue fills the cavity. (**E**) The LTKE peptide can bind hGBE1 via a number of hydrophobic and polar interactions (predicted hydrogen bonds in dotted lines).

Screening around the solvent exposed Ser329_mutant_ region in our hGBE1-Y329S structural model, the *ab initio* peptide design algorithm gave as best hit a Leu-Thr-Lys-Glu (LTKE) peptide among the six top scores (Supplementary Material, Table S3), in terms of favourable binding affinities and solubility. Molecular dynamics simulation of wild-type hGBE1, hGBE1-Y329S and LTKE peptide-bound hGBE1-Y329S models (Fig. 5A; Supplementary Material, Methods) corroborated our prediction that LTKE stabilizes the mutated enzyme. Modelling of the LTKE peptide onto our hGBE1-Y329S model suggests that the N-terminal Leu (position *i*) is the primary contributor to peptide-binding energy (Fig. 5B), with a calculated dissociation constant (K_d_) of 1.6 µm (Supplementary Material, Table S3). Replacement of Leu at position *i* with Ala (ATKE peptide) or with acetyl-Leu (Ac-LTKE peptide) was predicted to severely reduce peptide-binding energy (Supplementary Material, Fig. S7; Supplementary Material, Methods), strongly suggesting a specific mode of action for the LTKE peptide. In our LTKE-bound hGBE1-Y329S model, the Leu side-chain can penetrate the cavity formed by the p.Y329S mutation (Fig. 5C and D), recovering some of the hydrophobic interactions (e.g. with Phe327, Met362) offered by the wild-type tyrosyl aromatic ring, albeit with a different hydrogen bond pattern (Fig. 5E). The charged peptidyl N-terminus also hydrogen-bonds with Ser329_mutant_ and forms a salt bridge with Asp386. The peptidyl Thr at position *ii* hydrogen bonds to Asp386, whereas the side chains of Lys at position *iii* and Glu at position *iv* further provide long-range electrostatic interactions with hGBE1.

### Peptide rescue of hGBE1 p.Y329S

**Figure 6.**
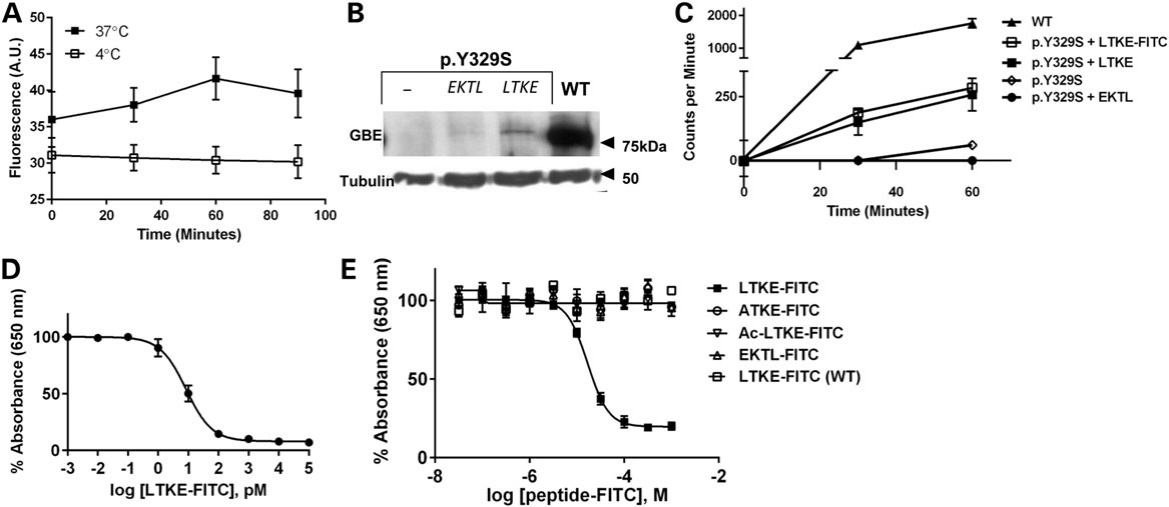
Peptide rescue of hGBE1 p.Y329S. (**A**) PBMCs isolated from APBD patients were incubated with FITC-labelled LTKE peptides at 37°C or 4°C. At the indicated times, intracellular peptide uptake was determined by flow cytometry. (**B**) Isolated PBMCs from an APBD patient (Y329S) or a control subject (WT) were incubated overnight with or without the peptides indicated (20µm). Lysed cells were subjected to SDS–PAGE and immunoblotting with anti-GBE1 and anti-α-tubulin (loading control) antibodies. (**C**) Isolated PBMCs treated as in (A) were assayed for GBE activity based on (27). (**D**) Standard curve showing displacement of solid phase FITC by soluble LTKE-FITC. Curve was fit by non-linear regression using the four-parameter logistic equation: % Absorbance (650) = Bottom + (Top-Bottom)/(1+10^((logEC50-log[LTKE-FITC])*Hillslope), where Bottom = 7.996, Top = 100, EC50 = 8.460, Hillslope = *−*1.015. *R*^2^ = 0.9934. (**E**) FITC-labelled peptide competition experiment. Curve fitting, using the homologous one-site competition model, was found for APBD patient cells competed with LTKE-FITC. APBD patient cells competed with control peptides, or wild-type cells competed with LTKE-FITC did not demonstrate competitive binding of LTKE-FITC. The competition model equation is: % Absorbance (650) = (Bmax*[LTKE])/([LTKE]+peptide-FITC (nM) + Kd (nM)), where *B*_max_ = 5229 nm, [LTKE] = 316 nm, K_d_ = 18 000 nM, Bottom = 13.24 nm. *R*^2^ = 0.9458. In all experiments, cells from *n* = 3 different APBD patients (or control unaffected subjects) were used. Error bars indicate SEM.

We evaluated the potential of the LTKE peptide to rescue the destabilized mutant protein *in vivo*, by testing it in APBD patient cells harbouring the p.Y329S mutation*.* To confirm that the peptide is internalized into cells, we determined its sensitivity to uptake temperature in peripheral blood mononuclear cells (PBMCs) and observed a time-dependent increase in the uptake of the C-terminal fluorescein isothiocyanate (FITC)-labelled peptide (LTKE-FITC) at 37°C but not 4°C, suggesting it is actively transported into cells (Fig. 6A). These peptide levels were sufficient to partially rescue mutant p.Y329S protein level *in vivo* as determined by Western blot analysis (Fig. 6B). Pre-incubation of PBMCs with the LTKE peptide resulted in detectable mutant GBE1 protein, which was absent when the ‘reverse peptide’ (EKTL) was used, or in patient-derived cells with no peptide treatment. More importantly, the LTKE and LTKE-FITC peptides enhanced GBE1 activity by 2-fold, compared with untreated or EKTL-treated mutant cells (>15% of unaffected control) (Fig. 6C). As these ameliorating effects of LTKE were sequence specific, we conjecture that they arose from the predicted hGBE1-Y329S binding model described in Figure 5, although attempts to prove this directly *in vitro* were hampered by difficulty in obtaining purified recombinant mutant protein (Fig. 4D). We resolved this by applying the hapten immunoassay (30) (Fig. 6D and E), to show that the LTKE-FITC peptide, but not the FITC-labelled control peptides ATKE, Ac-LTKE and EKTL with predicted inferior binding to hGBE1-Y329S model (Supplementary Material, Fig. S7), were able to out-compete LTKE binding in patient skin fibroblasts. This competitive binding of LTKE, specific to mutant cells and to the peptide amino acid sequence, clearly indicates the binding specificity of the LTKE peptide towards hGBE1 p.Y329S. The apparent K_d_ of peptide binding determined by the hapten immunoassay was 18 µm (Fig. 6E), within the range of error from the calculated K_d_ (1.6 µm; Supplementary Material, Table S3). Collectively, our data suggest that the LTKE peptide can potentially function as a stabilizing chaperone for the mutant p.Y329S protein.

### Concluding remarks

In this work, we combined structural, biochemical and cellular approaches to demonstrate for the first time that a GBE1 mutation can result in protein destabilization, lending support to the emerging concept, among many metabolic enzymes, that mutation-induced protein destabilization could play a causative role in disease pathogenesis (31). In this hypothesis, mutant destabilized proteins may misfold or aggregate and thus are subsequently degraded by the quality control machinery in the cell. Pharmacologic approaches to stabilize the partial misfolding using small molecule chaperones attempt to divert the mutant polypeptide from degradation pathways and deliver it to its native subcellular destination, ideally allowing a sufficient recovery of physiological function to prevent the disease state (28). Here, we provide proof of principle for use of a small peptide as chaperone therapy in APBD, showing that the LTKE peptide can rescue GBE1 mutant activity to 10–15% of wild type. We propose that the LTKE peptide binds to mutant GBE1 possibly in a co-translational manner, akin to the binding of cellular chaperones to nascent polypeptide chains during protein synthesis (32), thereby allowing peptide access to the mutation-induced cavity as the protein is being folded in the cell. In some metabolic disorders [e.g. lysosomal storage diseases, (33)], a 10–15% recovery of mutant enzyme activity was sufficient to ameliorate disease phenotypes. Specifically, in APBD, where patients homozygous for the p.Y329S mutation reportedly have GBE activity up to even 18% (34), a further 2-fold increase in activity with the peptide may be clinically significant, especially in conjunction with other therapies, as heterozygous carriers with only 50% GBE activity are non-symptomatic (27). Additionally, a peptide-mediated 10–15% improvement of GBE activity from null (e.g. p.F257L in GSDIV) might change a fatal childhood disease such as GSDIV, to a relatively more tolerable adult onset disease such as APBD.

In general, we believe small peptide-based therapy has a promising therapeutic potential: It has the benefits of low toxicity, low production costs and the possibility of incorporation into gene therapy, particularly useful in chronic conditions such as APBD. Nevertheless, peptides also have inherent disadvantages, such as poor oral bio-availability and low serum half-lives, which must be considered in future clinical applications. In summary, with a recombinant expression system and 3D structural information now available for human GBE1, a systematic, pharmacophore-based high-throughput screening regime using activity and stabilization as readout could be implemented in the future, to search for pharmacological chaperones that can target GBE1 as potential treatment for GSDIV and APBD.

## Material and Methods

### Recombinant hGBE1 production, crystallization and characterization

DNA fragment encoding aa 38–700 of human GBE1 (hGBE1_trunc_) was amplified from a cDNA clone (IMAGE: 4574938) and subcloned into the pFB-LIC-Bse vector (Gen Bank accession number EF199842) in frame with an N-terminal His_6_-tag and a TEV protease cleavage site. Full-length hGBE1 was constructed in the pFastBac-1 vector, from which the hGBE1-Y329S mutant was generated by two sequential PCR reactions using Exact DNA polymerase (5 PRIME Co, Germany). hGBE1 protein was expressed in insect cells in Sf9 media (Life technologies) and purified by affinity (Ni-NTA; Qiagen) and size exclusion (Superdex200; GE Healthcare) chromatography. hGBE1 was crystallized by vapour diffusion at 4°C. Diffraction data were collected at the Diamond Light Source. Phases for hGBE1 were calculated by molecular replacement. Atomic coordinates and structure factors have been deposited in the PDB with accession codes 4BZY, 5CLT and 5CLW.

### Peptide design, synthesis and uptake

Using Pepticom's proprietary software and its *ab initio* peptide design algorithm, a Leu-Thr-Lys-Glu (LTKE) peptide was selected for synthesis (GL Biochem, China). The effect of LTKE peptide was tested *in vivo* in peripheral blood mononuclear cells (PBMCs) collected from a healthy donor and APBD patients [approved by the Hadassah–Hebrew University Medical Center Institutional Review Board according to The Code of Ethics of the World Medical Association (Declaration of Helsinki)]. GBE1 activity was assayed as described (27). Cellular uptake of FITC-labelled peptide was measured by flow cytometry. Detailed method information is provided in Supplementary Materials, Methods.

### Hapten immunoassay for establishing competitive binding

Binding of peptides to hGBE1 p.Y329S in intact fibroblasts was assessed by competitive hapten immunoassay (30). Specific assay conditions are described in Figure 6's legend. In brief, a standard curve was first generated to show that the immunoreactive LTKE-FITC peptide in solution can compete for HRP-conjugated FITC antibody (Jackson Laboratories, West Grove, PA, USA) binding with solid phase FITC. To generate the standard curve, plates coated overnight with 12.5ng/ml BSA-FITC were incubated for 1h at room temperature with an HRP-conjugated anti-FITC antibody pretreated for 2h with different concentrations of LTKE-FITC. The HRP substrate tetra-methyl benzidine (TMB) was added for 0.5h, and absorbance at 650nm was measured by the DTX 880 Multimode Detector (Beckman Coulter, Indianapolis, IN, USA). Once competitive binding of the HRP-anti-FITC antibody was established by the standard curve, either APBD skin fibroblasts expressing only hGBE1 p.Y329S or control skin fibroblasts expressing only wild-type hGBE1 were incubated with 316 nM LTKE peptide [about a log concentration below the model-predicted 1.6 µm affinity of LTKE towards hGBE1 p.Y329S (Supplementary Material, Table S3)] and varying concentrations of the FITC-labelled LTKE and control peptides. The concentration range was designated to test displacement of the unlabelled LTKE peptide from hGBE1 p.Y329S, which generates an immunoreactive FITC hapten capable of competing with solid-phase FITC in the assay. This competition decreases the HRP-conjugated FITC antibody binding to solid-phase FITC. To perform this competition assay, plates coated with BSA-FITC as above were incubated for 1h with an HRP-conjugated anti-FITC antibody pretreated for 2h with cell lysates of fibroblasts from APBD patients homozygous for the GBE p.Y329S mutation and control patients. These fibroblasts were in turn treated for 2h with 316 nm LTKE and different concentrations of FITC-labelled LTKE and control peptides. TMB was then added and absorbance at 650nm measured.

## Supplementary material

Supplementary Material is available at *HMG* online.

## Funding

The Structural Genomics Consortium is a registered charity (number 1097737) that receives funds from AbbVie, Boehringer Ingelheim, the Canada Foundation for Innovation, the Canadian Institutes for Health Research, Genome Canada, GlaxoSmithKline, Janssen, Lilly Canada, the Novartis Research Foundation, the Ontario Ministry of Economic Development and Innovation, Pfizer, Takeda, and the Wellcome Trust (092809/Z/10/Z). W.W.Y. is further supported by a gift donation from the APBD Research Foundation. O.K. is supported by grants from the Association Francaise contre Myopathy and the APBD Research Foundation. Funding to pay the Open Access publication charges for this article was provided by Wellcome Trust.

## Supplementary Material

Supplementary Data
