# Supplementary material for: Structural basis of glycogen branching enzyme deficiency and pharmacologic rescue by rational peptide design
Source: Hum Mol Genet. 2015 Jul 21;24(20):5667–76. doi: 10.1093/hmg/ddv280 (PMC4581599; doi:10.1093/hmg/ddv280)
Supplement: Supplementary Data [file supp_24_20_5667__index.html]

Structural basis of glycogen branching enzyme deficiency and pharmacologic rescue by rational peptide design — Structural basis of glycogen branching enzyme deficiency and pharmacologic rescue by rational peptide design — Supplementary Data 

# Structural basis of glycogen branching enzyme deficiency and pharmacologic rescue by rational peptide design

## Supplementary Data

Supplementary Data

- Supplementary Data - Docx file
