# Supplementary material for: Structural basis of glycogen branching enzyme deficiency and pharmacologic rescue by rational peptide design
Source: Hum Mol Genet. 2015 Jul 21;24(20):5667–76. doi: 10.1093/hmg/ddv280 (PMC4581599; doi:10.1093/hmg/ddv280)
Supplement: Supplementary Data [file supp_ddv280_ddv280supp_data.docx]

**Structural basis of glycogen branching enzyme deficiency and pharmacologic rescue by rational peptide design**

D. Sean Froese^1,†,§^, Amit Michaeli^2,†^, Thomas J. McCorvie^1,†^, Tobias Krojer^1^, Meitav Sasi^3^, Esther Melaev^3^, Amiram Goldblum^2,4^, Maria Zatsepin^2^, Alexander Lossos^3^, Rafael Álvarez^5^, Pablo V. Escribá^5^, Berge A. Minassian^6^, Frank von Delft^1^, Or Kakhlon^3,*^, Wyatt W. Yue^1,*^

^1^Structural Genomics Consortium, Nuffield Department of Clinical Medicine, University of Oxford, UK OX3 7DQ

^2^Pepticom LTD., Jerusalem, Israel

^3^Department of Neurology, Hadassah-Hebrew University Medical Center, Ein Kerem, Jerusalem, Israel

^4^Institute for Drug Research, The Hebrew University of Jerusalem, Jerusalem, Israel

^5^Department of Biology, University of the Balearic Islands, E-07122 Palma de Mallorca, Spain

^6^Program in Genetics and Genomic Medicine, The Hospital for Sick Children, University of Toronto

^†^D.S.F., A.M., T.M. contributed equally to this work.

*O.K., W.W.Y. contributed equally to this work, and to whom correspondence should be addressed:

W.W.Y. ([wyatt.yue@sgc.ox.ac.uk](mailto:wyatt.yue@sgc.ox.ac.uk)),

O.K. ([ork@hadassah.org.il](mailto:ork@hadassah.org.il))

^§^D.S.F. present address: Division of Metabolism and Children’s Research Center, University Children’s Hospital, Zurich CH-8032, Switzerland

**Supplementary Material**

**Supplementary Methods**

**Fig. S1.** Constructs of hGBE1 attempted for recombinant expression

**Fig. S2.** Interaction of oligosaccharides with CBM48 domain

**Fig. S3.** GH13 amylolytic structures bound with active site oligosaccharides

**Fig. S4.** GBE1 sequence motifs.

**Fig. S5.** The two-step catalytic mechanism proposed for the hGBE1 branching reaction

**Fig. S6.** Amino acid conservation of *GBE1* missense mutation sites

**Fig. S7.** Binding prediction of LTKE and control peptides

**Table S1.** Data collection and refinement statistics

**Table S2.** List of *GBE1* missense mutations

**Table S3.** Peptide ensemble analysis

**Supplementary References**

**SUPPLEMENTARY METHODS**

***Cloning expression and purification of hGBE1***

For structural studies, a DNA fragment encoding aa 38-700 of human GBE1 (hGBE1_trunc_) was amplified from a cDNA clone (IMAGE: 4574938) and subcloned into the pFB-LIC-Bse vector (Gen Bank accession number EF199842) in frame with an N-terminal His_6_-tag and a TEV protease cleavage site. Protein was expressed in High Five Insect Cells (Life technologies) in Sf9 media (Life technologies) supplemented with 1% FCS. 120 hours post infection the cultures were harvested and centrifuged for 20 min at 1500 x *g*. Cell pellets were dissolved in lysis buffer (50 mM HEPES, pH 7.4; 500 mM NaCl; 5% glycerol; 10 mM imidazole, pH 7.4) and lysed by sonication (Vibra-Cell) and homogenization (Emulsiflex C5). hGBE1 protein in the clarified supernatant was purified by affinity (Ni-NTA; Qiagen) and size-exclusion (Superdex200; GE Healthcare) chromatography. Pure protein fractions were concentrated to 16.5 mg/ml and stored in buffer containing: 50 mM HEPES, pH 7.4; 500 mM NaCl; and 5% glycerol at -80°C.

For biochemical studies, cDNA encoding full-length hGBE1 was produced by PCR using primers that introduced a C-terminal non-cleavable His_6_-tag and EcoRI (5’ end) and HindIII (3’ end) restriction sites by PCR amplification. The DNA generated was inserted into the pFastBac-1 plasmid, sequenced twice (both DNA strands) and introduced in *E. coli* XL1-blue for amplification. The hGBE1 p.Y329S mutant was generated from this recombinant plasmid by two sequential PCR reactions using Exact DNA polymerase (5 PRIME Co, Germany). The wild-type (WT) and p.Y329S hGBE1 cDNAs cloned in pFastBac-1 were introduced in *E. coli* DH10Bac competent cells, which contain the AcNPV (*Autographa califormica* nuclear polyhedrosis virus). The cDNAs were transferred from pFastBac-1 to the AcNPV bacmid by site-specific transposition. Finally, AcNPV bacmids containing full-length WT or p.Y329S GBE1 were purified using the Plasmid Midi kit (Qiagen) and introduced into Sf9 insect cells Cellfectin (Invitrogen) as transfection agent. Full-length hGBE1 (WT and mutant) was purified similarly as with hGBE1_trunc_.

***Crystallization, structure determination and model building***

Crystals of apo-hGBE1 were grown by vapor diffusion at 4°C, in sitting drops consisting of 50 nl protein and 100 nl well solution, equilibrated against well solution containing 0.05-0.2 M sodium-succinate and 17-22% PEG 3350 (v/v). Crystals of oligosaccharide-bound hGBE1 were grown in similar conditions using purified protein (12 mg/ml) pre-incubated with 5 mM ligand (acarbose, ACR or maltoheptaose, Glc7). Crystals were mounted in the presence of 25% (v/v) ethylene glycol and flash-cooled in liquid nitrogen. Diffraction data was collected at the Diamond Light Source beamlines I04 (apo), I02 (ACR) and I04-1 (Glc7), and processed using the CCP4 program suite ([1](#_ENREF_1)).

Initial phases of hGBE1-apo were calculated by molecular replacement with PHASER ([2](#_ENREF_2)), using the rice BE structure (PDB 3AMK) as starting model. ARP/wARP ([3](#_ENREF_3)) was subsequently used for automated model building, followed by iterative cycles of PHENIX ([4](#_ENREF_4)) refinement and model building with COOT ([5](#_ENREF_5)). The final models comprise three protomers in the asymmetric unit with essentially identical conformations (C^α^-rmsd 0.22 Å), all harboring a disordered loop region (A:368-377, B:368-383, C:368-383). Phases for the hGBE1-ACR and hGBE1-Glc7 structure were derived by difference Fourier method with the hGBE1-apo structure. The hGBE1-ACR and hGBE1-Glc7 structures, adopting essentially identical conformations as *apo* hGBE1 (C^α^-RMSD ~0.12 Å), revealed significant difference density for all saccharide units in all three protomers. The directionality of ACR within the model is guided by difference density features accounting for the lack of 6-hydroxyl group in the 4,6-dideoxyglucose moiety. All protein residues that contact the oligosaccharides are well defined in the electron density map. Attempts to soak ACR/Glc7-cocrystallized hGBE1 crystals with further ligands did not yield discernible sugar electron density at the active site. Atomic coordinates and structure factors have been deposited with the Protein Data Bank under the accession codes 4BZY (hGBE1-apo), xxxx (hGBE1-ACR) and xxxx (hGBE1-Glc7).

***Peptide design and synthesis***

Based on the wild-type hGBE1 structure, the Y329S mutation was modelled with a ‘rigid backbone’ limitation using Pepticom’s proprietary software. A 17 Å grid was constructed at a 1 Å resolution in the solvent exposed region around position 329. Pepticom’s *ab initio* peptide design algorithm (Michaeli A, PhD thesis, Hebrew University of Jerusalem; to be published) was used to search for possible peptides within the grid which show favorable calculated binding affinities to the mutated GBE protein and reasonable solubility. The algorithm was supplemented by the Risk Adjusted Design algorithm (to be published separately), to generate a binding candidate ensemble. From the solution ensemble, a Leu-Thr-Lys-Glu (LTKE) peptide was selected for synthesis due to its calculated micromolar binding affinity, small size and the presence of a cationic lysine residue, which could increase the probability of cell membrane penetration *via* active transport. The peptide was synthesized by GL Biochem (Shanghai, China) using solid phase synthesis at a 98% level of purity.

***Binding prediction model for control peptides***

The three control peptides for the LTKE binding models include: ‘ATKE’, an Ala mutant of Leu at position *i*, calculated to be the most contributing residue to the peptide binding energy (Fig. 5B); ‘Ac-LTKE’, an N-terminal acetylated LTKE peptide, predicted by the LTKE binding model to both cause steric clashes and prevent the N-terminus hydrogen bonds; and ’EKTL’, a peptide where the LTKE sequence is reversed. The control peptides were then docked using Schordinger’s Glide docking module with the following parameters: VdW scaling factor - 1; Partial Vdw charge cuttoff – 0.25; Docking grid using ligand centroid; rotatble OHs for Ser290, Thr318, Ser324, Ser329, Ser330, Thr360 and Ser361; SP-peptide docking with flexible ligand sampling; N-inversion; Epik state penalites; distance dielectric constant of 2; max minimization steps 100; ring sampling 2.5 Kcal/mol. The top scoring conformations for each peptide were then compared. All three control peptides were predicted to have reduced binding affinity when compared to the LTKE peptide, with ATKE showing a binding energy suggestive of millimolar binding affinity (eventually did not show binding, Fig. 6E) and Ac-LTKE and EKTL showing an energy suggestive of non-binding. While the backbones of LTKE and ATKE remained inside the cavity formed by the Y329S mutation, the Ac-LTKE and EKTL peptides were located outside the cavity.

***Molecular Dynamics simulation of hGBE1, hGBE1-Y329S and LTKE-bound hGBE1-Y329S***

Structure of the wild-type human GBE1 (WT) (GBE1; PDB: 4BZY) and of Oryza sativa starch branching enzyme I (SBE1; PDB: 3AMK; 54% identity to hGBE1) were retrieved. The mutant hGBE1-Y329S model was created by modification of the WT structure with and without the LTKE peptide. Missing hydrogens were added, side chains optimized and fixed and bond orders were assigned for all the structures using Protein Preparation Tool of Maestro v10.1 (Schrodinger, Inc).

A relatively long loop segment of 18 amino acids (aa 366-383) within the crystallographic structure of the WT was missing. Such long loops are very hard and time consuming for accurate reconstruction by computational methods due to their high flexibility and length. For this reason and due to high identity of the GBE1 and SBE1 structures, and mainly their aa366-383 regions, chimeric structures of WT and the Y329S mutant were created by splicing the missing loop segment from the SBE1 structure (identity 61%, similarity 83% by MSV Maestro tool). Automatic minimization within 6 bonds region around the splicing sites was performed (VSGB implicit solvent; dielectric constant of 80; RMS of 0.01 Kcal/Mol/A; conjugated gradient changing to truncated newton for small gradients; 2 iterations).

Molecular dynamics (MD) was performed using explicit water model, in a similar conditions as was reported recently for mutant comparison ([6-10](#_ENREF_6)). All the atoms, including water were represented explicitly. The protein structures were inserted by TIP3P ([11](#_ENREF_11), [12](#_ENREF_12)) orthorhombic solvent model with a minimum solute-wall distance of 12Å, in 0.15M NaCl. The system was relaxed in the NTP ensemble using Langevin Thermostat method (300K, 5ps) and Langevin Barostat method (1.0135 bar, 10ps) with isotropic integration. MD was performed using Desmond (Schrodinger, Inc). Equilibrium for 500ps at NPT 300K, and a run for 25ns based on similar dynamics run published (steps: 2fs - bonded, near; 6fs – far; frames - 1.2ps) with initial Boltzmann distributed randomized velocities. Such long MD was performed due to the main interest for investigation of the stabilization of mutant by LTKE peptide in comparison with WT protein. Such a comparison requires long-term run and overall backbone movement for the proteins. During the MD simulation the Smooth Particle Mesh Ewald (SPME; tolerance 10A) method was employed to deal with long-range electrostatics interactions ([13](#_ENREF_13), [14](#_ENREF_14)), while short range Coulomb electrostatics were calculated with potential tapering between 8-10A. The atomic coordinates were saved each 1.2ps (integration of 4.8).

The dynamics conformation of each 0.5ns of the three structures were superimposed with the initial backbone of the crystal structure in order to compare the Root Mean Squared Deviations of the backbone (RMSD) as a representation of structures stability ([7](#_ENREF_7)).

***Peptide uptake***

The effect of designed peptides on GBE activity was tested in peripheral blood mononuclear cells (PBMCs) collected from a healthy donor and APBD patients (approved by the Hadassah-Hebrew University Medical Center Institutional Review Board according to The Code of Ethics of the World Medical Association (Declaration of Helsinki)). PBMCs were isolated by a Ficoll-based density gradient as described ([15](#_ENREF_15)). Briefly, whole blood diluted 1:1 with PBS was added on top of Ficoll (Axis-Shield, Oslo, Norway) and spun down at 800 x g for 20 min. The interphase between plasma and Ficoll-erythrocytes contained PBMCs and was collected, washed 3 times with PBS and suspended at 2x10^6^ cells/ml in PBS supplemented with 2% fetal bovine serum (FBS). FITC-labelled peptides (20 µM) were then added to the PBMCs suspensions and fluorescence was measured at the indicated time intervals (10,000 events per time point), by a FC 500 flow cytometer (Beckman Coulter) using the 488 excitation laser line and FITC (FL-1) detector.

***GBE activity and expression***

Isolated PBMCs were incubated overnight in RPMI medium with 10% FBS alone or with 20 µM of peptides. Cells were harvested and assayed for either GBE activity or protein levels. GBE activity was assayed as previously described ([15](#_ENREF_15)) and GBE levels was assayed by sodium dodecyl sulphate polyacrylamide gel electrophoresis (SDS-PAGE) and immunoblotting using anti-GBE1 (Abnova, Taiwan) and anti-alpha-tubulin (Abcam, UK) antibodies.

**Figure S1**


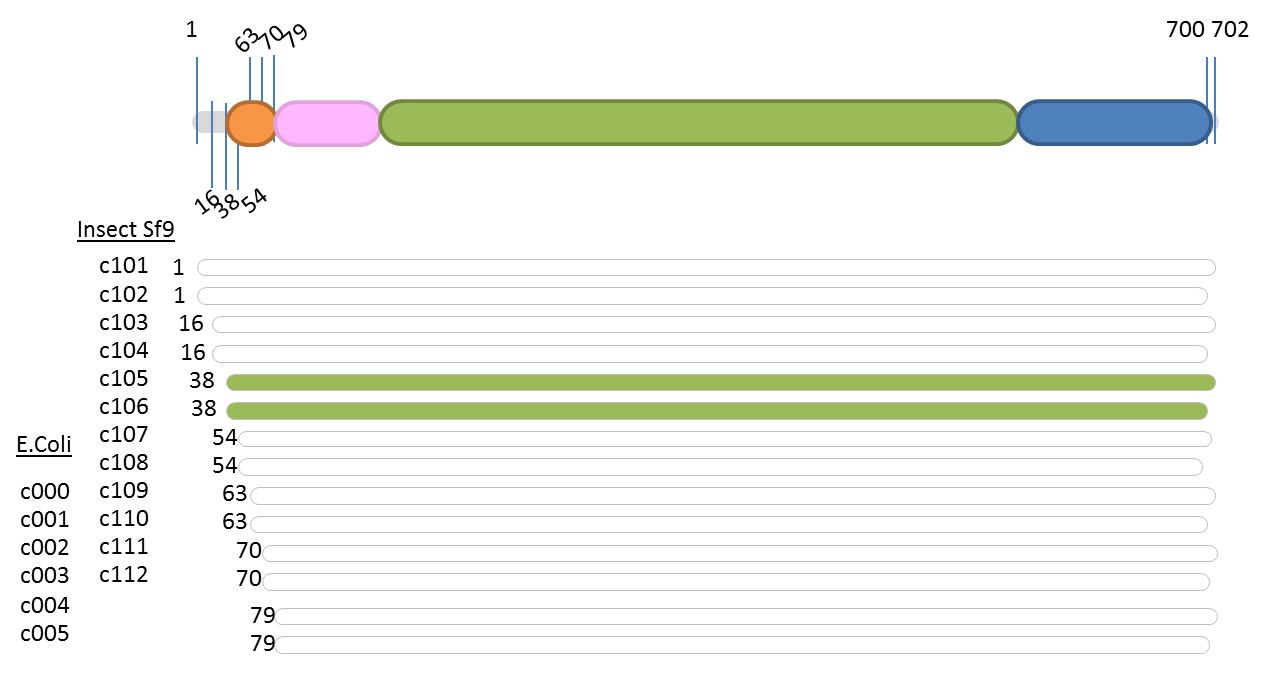


**Fig. S1.** Constructs of hGBE1 attempted for recombinant expression. Highlighted in green are two constructs that gave milligram quantities of soluble protein when expressed in litre scale. The orange, pink, green and blue bars denote the helical segment, CBM48 domain, catalytic domain, and C-terminal amylase domain respectively.

**Figure S2**


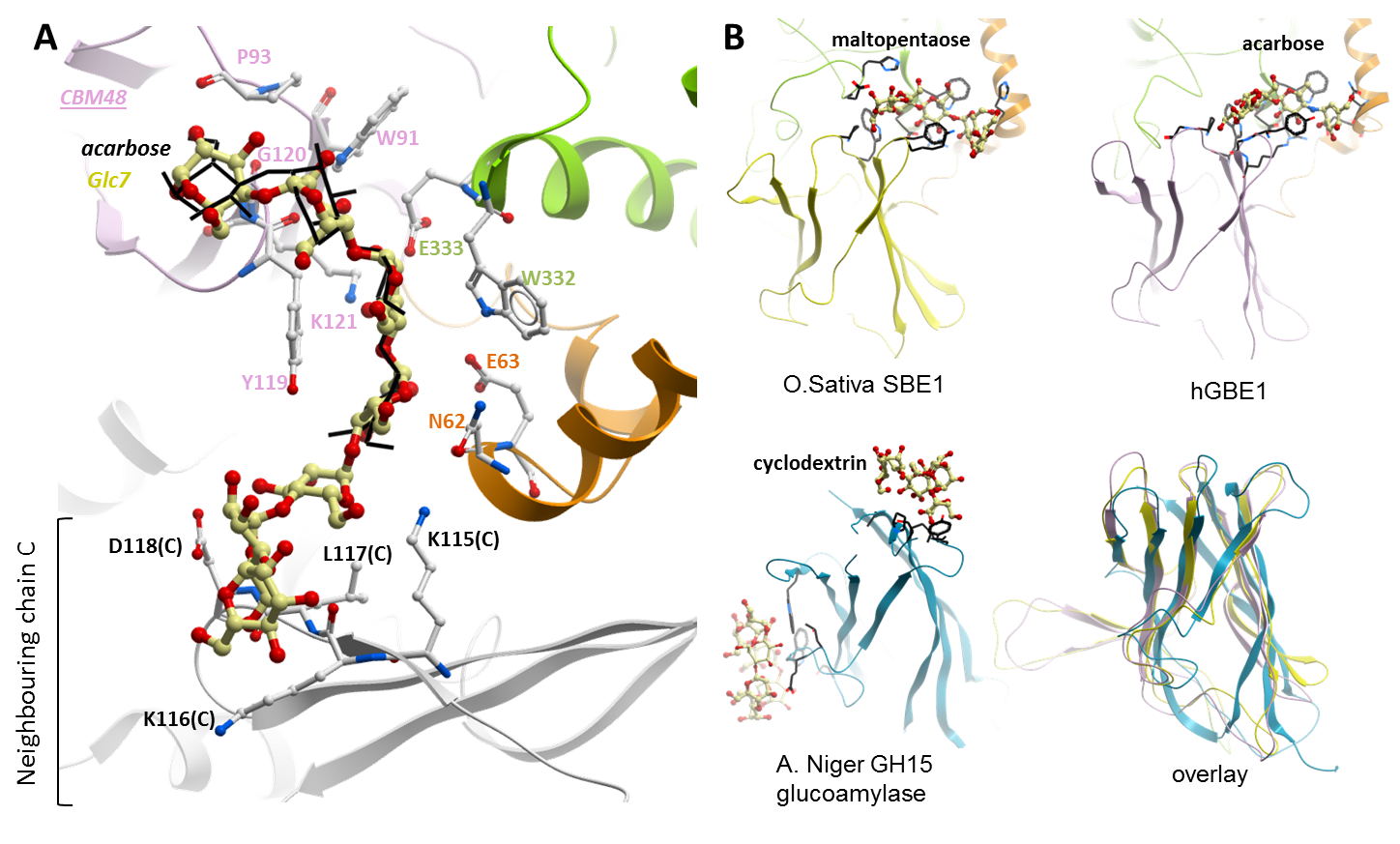


**Fig. S2.** Interaction of oligosaccharides with CBM48 domain. (*A*) Binding mode of maltoheptaose in the hGBE1-Glc7 structure, coloured in the same scheme as Fig. 2C. The orientation of acarbose is also shown as an overlay from the hGBE1-ACR structure. (*B*) Comparison of oligosaccharide binding mode of CBM48 modules from: the *O.sativa* SBE1 structure complexed with maltopentaose (PDB 3vu2, *top left*), hGBE1 structure complexed with acarbose (this study, *top right*) and *A. Niger* GH15 glucoamylase structure complexed with cyclodextrin (PDB 1ac0, *bottom left*). The three CBM48 modules are superimposed in the *bottom right* panel.

**Figure S3**


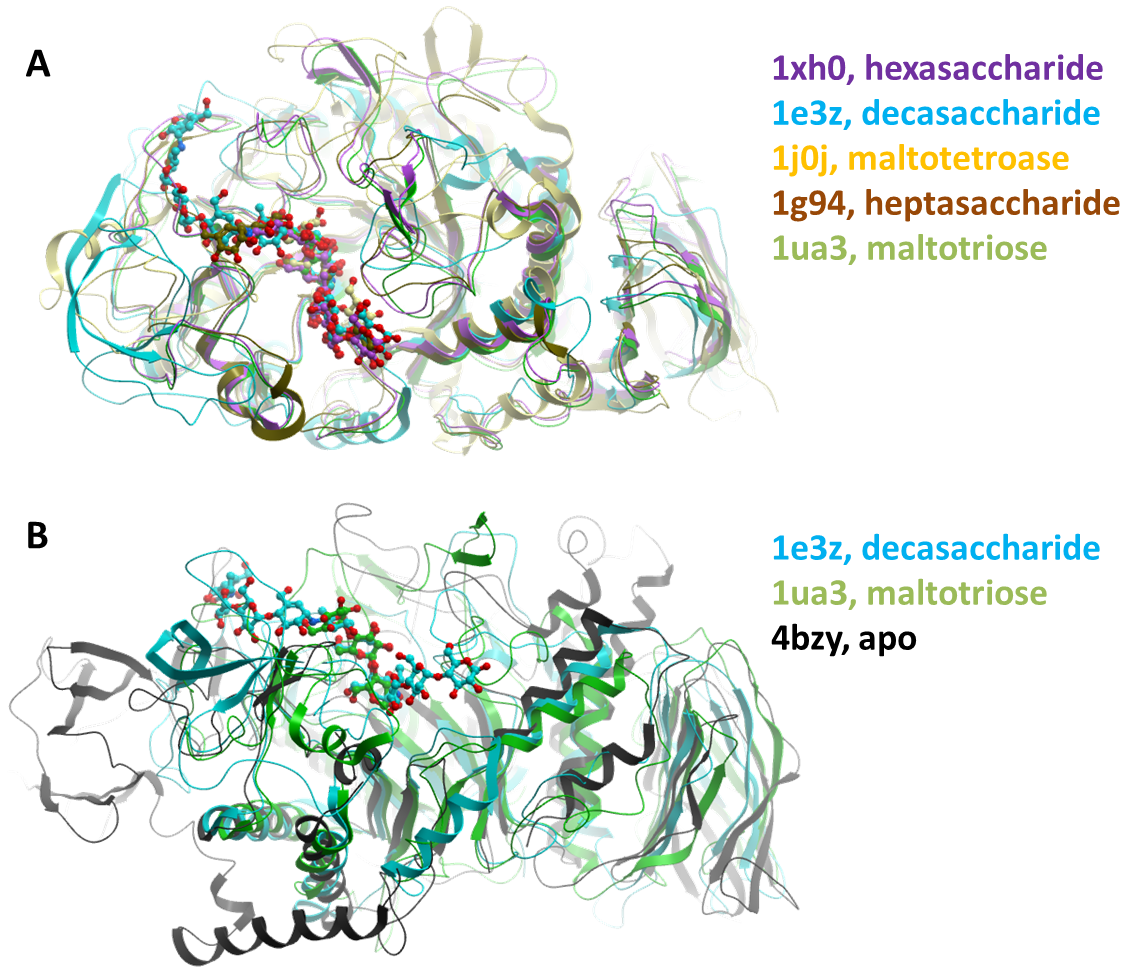


**Fig. S3.** GH13 amylolytic structures bound with active site oligosaccharides. (*A*) Structural superposition of human pancreatic α-amylase bound with an acarbose-derived hexasaccharide (PDB 1xh0, purple), a chimeric α-amylase complex from *B. amyloliquefaciens* and *B. licheniformis* bound with a decasaccharide (1e3z, blue), *B. stearothermophilus* TRS40 neopullulanase bound with maltotetraose (1j0j, orange), *P. haloplanctis* α-amylase bound with a heptasaccharide (1g94, brown), and pig pancreatic α-amylase bound with maltotriose (1ua3, green). (*B*) Structural superposition of hGBE1-apo (4bzy, black) overlayed with 1e3z and 1ua3 structures. In both panels, each oligosaccharide chain is in stick representation and its carbon atoms are coloured corresponding to the respective protein ribbon. The view in panel *B* is rotated 45^o^ along the y-axis from that of panel *A*.

.

**Figure S4**


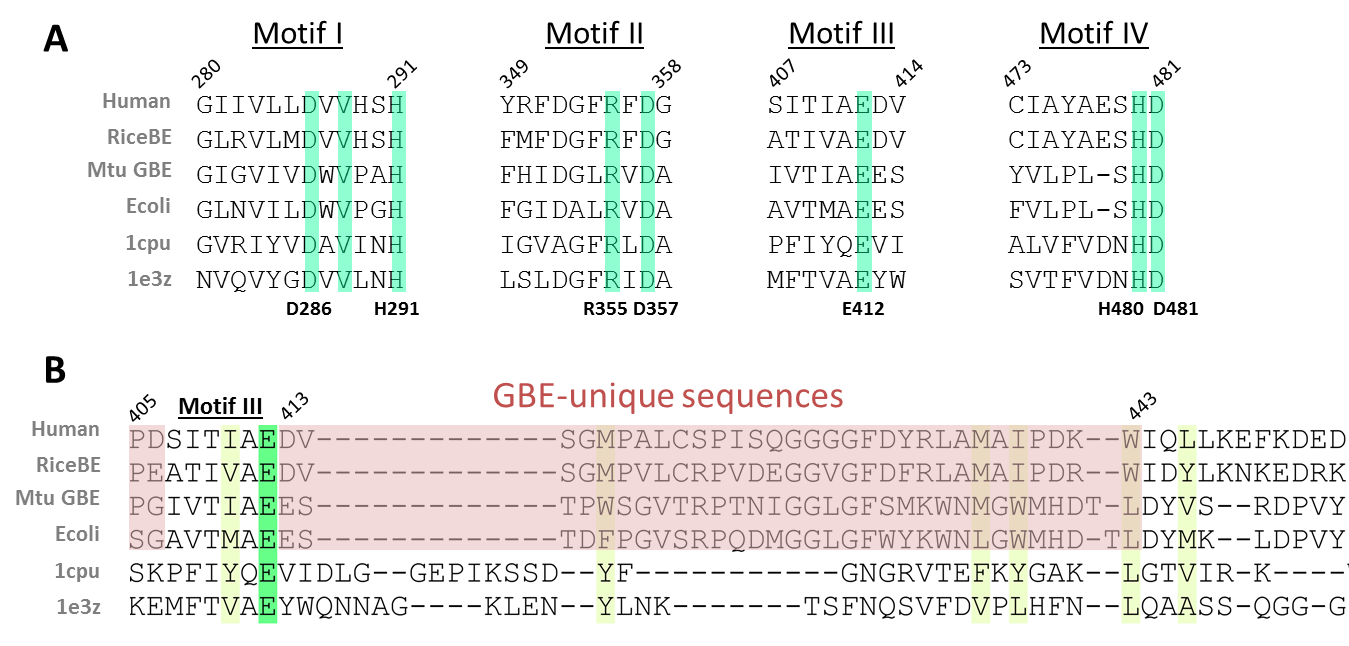


**Fig. S4.** GBE1 sequence motifs. (*A*) Alignment of sequences constituting the four conserved motifs among the GH13 family of enzymes, highlighting the strictly conserved seven amino acids that form the “-1” subsite. The annotated sequences are branching enzymes from human, *O. sativa* (RiceBE), *M.tuberculosis* (Mtu GBE) and *E. coli*, as well as human pancreas α-amylase (1cpu) and the chimeric α-amylase complex from *B. amyloliquefaciens* and *B. licheniformis* (1e3z). (*B*) Sequence alignment of a ~30 amino acid stretch (pink) that is conserved among branching enzyme orthologues, but not among amylases within the GH13 family.

**Figure S5**


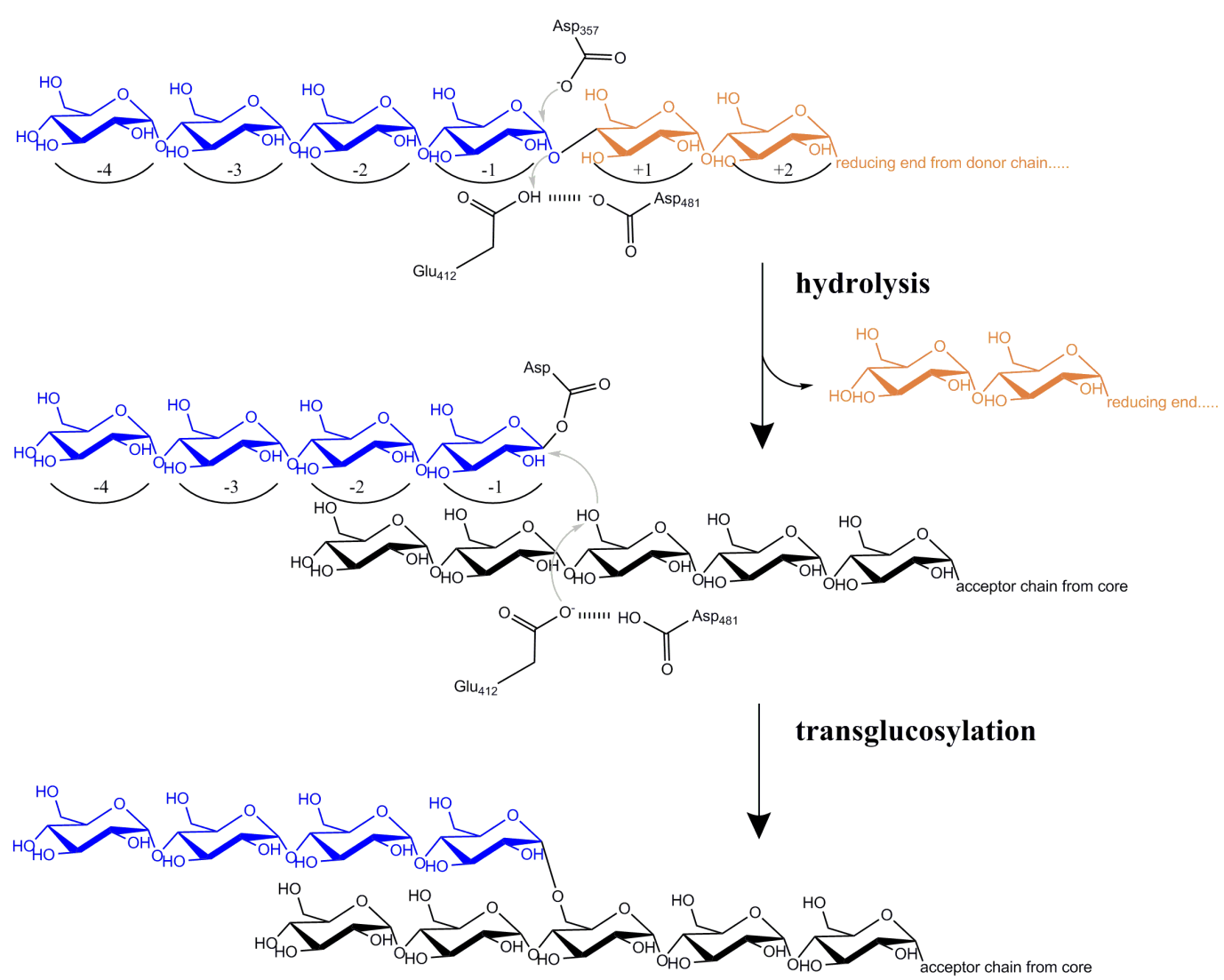


**Fig. S5.** The two-step catalytic mechanism proposed for the hGBE1 branching reaction. Sugar subsites are indicated by arcs, nucleophilic attacks by grey arrows, and hydrogen bonds by dashed lines. The donor and acceptor glucan chains are colored blue and black respectively.**Figure S6**


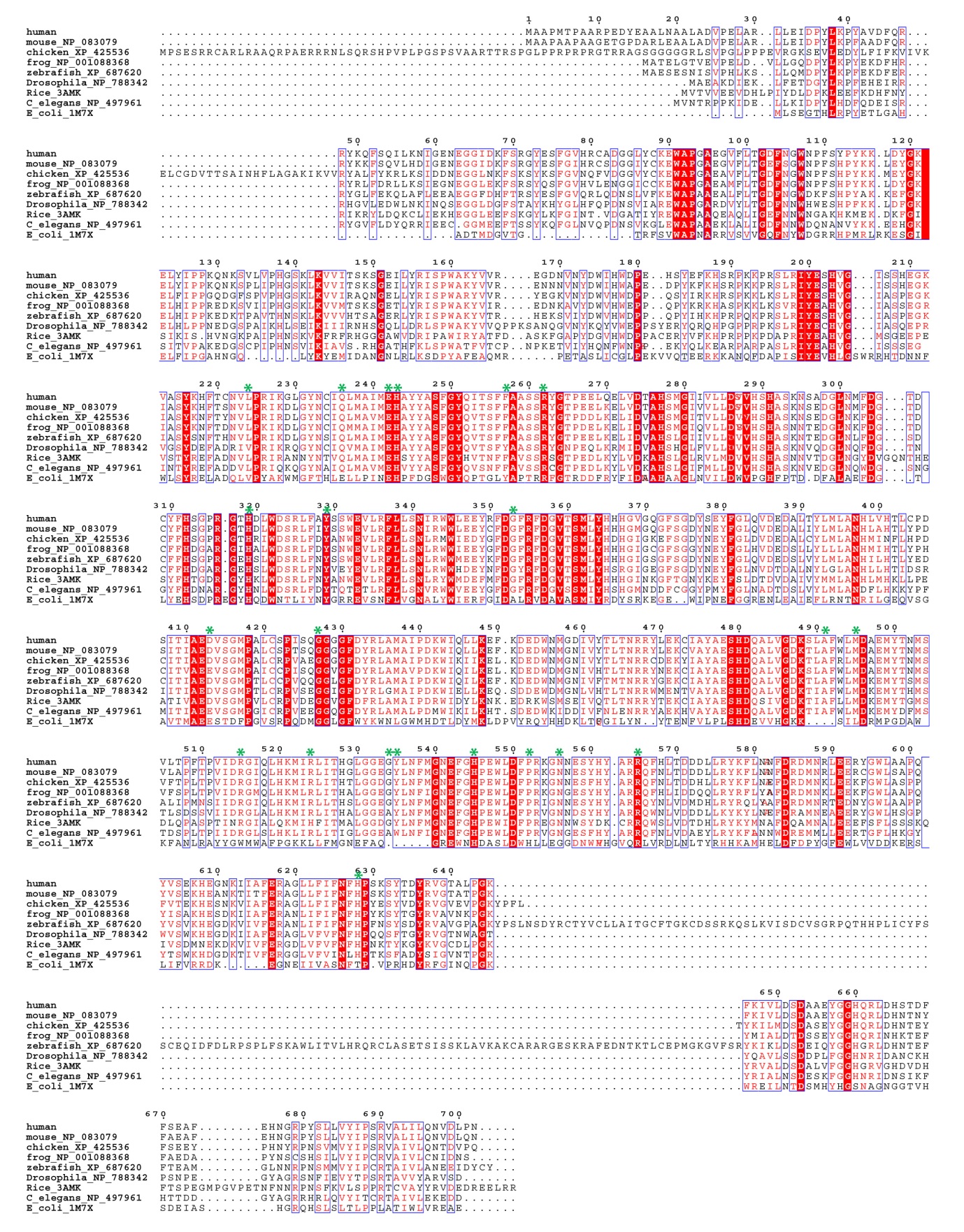
 **Fig. S6.** Amino acid conservation of *GBE1* missense mutation sites, marked as green asterisks. Identical amino acids are highlighted in red, conserved are in red font. Alignment was made using multalin ([16](#_ENREF_16)) and visualized by ESPript ([17](#_ENREF_17)).

**Figure S7**

**Fig. S7.** Binding prediction of LTKE and control peptides. Control peptides were designated ATKE, Ac-LTKE and EKTL. The peptides were then docked using the Glide module of Schrodinger’s software and re-scored using Pepticom’s energy function. The top scoring model for each peptide is shown in red (scoring function unit, kcal/mol)

**Table S1** Crystallography refinement statistics.

|  | hGBE1-apo | hGBE1-ACR | hGBE1-Glc7 |
| --- | --- | --- | --- |
| **Overall Description** |  |  |  |
| Pdb code | 4BZY | 5CLT | 5CLW |
| Ligands bound | - | ACR | Glc7 |
|  |  |  |  |
| **Data collection** |  |  |  |
| Beamline | Diamond I04-1 | Diamond I03 | Diamond I04 |
| Wavelength (Å) | 0.92 | 0.9795 | 0.9795 |
| Unit cell parameters (Å) | 117.3, 164.5, 311.3 | 116.8, 164.0, 311.7 | 116.7, 164.5 , 313.2 |
| α=β=γ(°) | 90 | 90 | 90 |
| Space group | C222_1_ | C222_1_ | C222_1_ |
| Resolution range (Å) | 91.3 – 2.75 (2.90-2.75) | 72.5 – 2.79 (2.86-2.79) | 313-2.80 (3.13-2.80) |
| Rmerge(%) | 0.174 (0.873) | 0.118 (0.697) | 0.153 (0.758) |
| I/sig(I) | 16.3 (2.0) | 10.0 (2.1) | 9.2 (2.1) |
| Completeness | 99.9 (100.0) | 99.7 (99.7) | 99.8 (99.8) |
| Multiplicity | 18.1 (7.8) | 3.8 (3.8) | 4.5 (4.7) |
|  |  |  |  |
| **Refinement** |  |  |  |
| Rcryst (%) | 0.1845 | 0.1937 | 0.1828 |
| Rfree (%) | 0.2251 | 0.2393 | 0.2152 |
| Wilson *B* factor (Å^2^) | 48.75 | 48.86 | 52.34 |
| Average total *B* factor (Å^2^) | 46.02 | 38.95 | 55.37 |
| Average ligand *B* factor (Å^2^) | n/a | 50.93 | 68.90 |
| Ligand occupancy | n/a | 1.00 | 1.00 |
| Rmsd bond length (Å) | 0.003 | 0.009 | 0.004 |
| Rmsd bond angle (°) | 0.759 | 1.253 | 0.81 |
| Ramachandran outliers (%) | 0.05 | 0.16 | 0.00 |
| Ramachandran favoured (%) | 98.06 | 97.64 | 97.96 |

Data for highest resolution shell are shown in parenthesis.

**Table S2.** List of *GBE1* missense mutations

| **Protein Change** | **DNA change** | **Exon** | **Disease phenotypes** | **Reference** |
| --- | --- | --- | --- | --- |
| p.L224P | c.671C>T | 5 | Nonprogressive hepatic; APBD | ([18](#_ENREF_18)) |
| p.Q236H | c.708G>C | 6 | Childhood neuromuscular (mild) | ([19](#_ENREF_19)) |
| p.E242Q | c.724G>C | 6 | APBD | ([20](#_ENREF_20)) |
| p.H243R | c.728A>G | 6 | Neonatal neuromuscular | ([21](#_ENREF_21)) |
| p.F257L | c.771T>A | 6 | Classic hepatic | ([18](#_ENREF_18)) |
| p.R262C | c.784C>T | 7 | Childhood neuromuscular (mild) | ([18](#_ENREF_18), [19](#_ENREF_19)) |
| p.H319R | c.956A>G | 7 | foetal akinesia deformation sequence | ([22](#_ENREF_22)) |
| p.H319Y | c.955C>T | 7 | APBD | ([23](#_ENREF_23)) |
| p.Y329S | c.986A>C | 8 | Nonprogressive hepatic, APBD | ([15](#_ENREF_15), [16](#_ENREF_16), [18](#_ENREF_18), [24](#_ENREF_24)) |
| p.Y329C | c.986A>G | 8 | APBD | ([20](#_ENREF_20)) |
| p.G353A | c.1058G>C | 8 | APBD | ([23](#_ENREF_23)) |
| p.D413H | c.1237G>C | 10 | APBD | ([25](#_ENREF_25)) |
| p.G427R | c.1279G>A | 10 | Classic hepatic | ([26](#_ENREF_26)) |
| p.A491Y | c.1471G>C | 12 | foetal akinesia deformation sequence | ([27](#_ENREF_27)) |
| p.M495T | c.1484T>C | 12 | Classic hepatic | ([28](#_ENREF_28)) |
| p.R515C | c.1543C>T | 12 | Classic hepatic | ([18](#_ENREF_18), [28](#_ENREF_28)) |
| p.R515H | c.1544G>A | 12 | APBD | ([29-32](#_ENREF_29)) |
| p.R524Q | c.1571G>A | 12 | APBD, classic hepatic | ([21](#_ENREF_21), [29](#_ENREF_29), [30](#_ENREF_30)) |
| p.G534V | c.1601G>T | 12 | APBD | ([25](#_ENREF_25)) |
| p.Y535C | c.1604A>G | 12 | Classic hepatic | ([28](#_ENREF_28), [33](#_ENREF_33)) |
| p.N541D | c.1623A>G | 13 | APBD | ([34](#_ENREF_34)) |
| p.H545R | c.1634A>G | 13 | Neonatal neuromuscular | ([21](#_ENREF_21)) |
| p.P552L | c.1655C>T | 13 | Classic hepatic | ([28](#_ENREF_28)) |
| p.N556Y | c.1666A>T | 13 | APBD | ([20](#_ENREF_20)) |
| p.R565Q | c.1694G>A | 13 | APBD | ([20](#_ENREF_20)) |
| p.H628R | c.1883A>G | 14 | Childhood neuromuscular | ([21](#_ENREF_21)) |

**Table S3.** Peptide ensemble analysis

| **Model** | **Binding Free Energy***  (kcal/mol, calculated) | **Expected Molar Dissociation Constant (Kd)**** | **Sequence** | **Fills The Y329S Space?** | **Model Description** |
| --- | --- | --- | --- | --- | --- |
| 1 | -13.46 | ${1.3x10}^{-7}$ | EKEPFEMFM | NO | Primarily long range electrostatics and hydrophobic interactions. |
| 2 | -10.00 | ${1.6x10}^{-6}$ | LTKE | YES | Hydrogen bond pattern combined with hydrophobic interactions. |
| 3 | -9.46 | ${2.4x10}^{-6}$ | SSKI | YES | Very similar model to 2, with lower calculated affinity and less optimal H-bond pattern. |
| 4 | -9.13 | ${3.1x10}^{-7}$ | MKWE | Partially | Primarily long range electrostatics and hydrophobic interactions. |
| 5 | -8.57 | ${4.6x10}^{-6}$ | KSLRKW | NO | Primarily long range electrostatics and hydrophobic interactions. |
| 6 | -8.26 | ${5.8x10}^{-6}$ | SDHRKMYEGR | NO | A helical model primarily composed of electrostatic interactions. |

*Calculated using Pepticom’s energy function, with the relationship to measured binding free energies: $\Delta Gmeasured=(0.44)(\Delta Gcalculated)-3.6$

(based on the calculated to measured linear regression of 55 peptide-protein and protein-protein complexes with similar characteristics to the design parameters, R^2^=0.47).

**Obtained using the equation: $Kd=e^{\Delta Gi/RT}$ where $\Delta Gi=\left( 0.44 \right)(\Delta Gcalculated)-3.6$ which serves as an estimate for the dissociation constant.

**Supplementary References**

1. CCP4 (1994) The CCP4 suite: programs for protein crystallography. *Acta Crystallogr. D Biol. Crystallogr.* 50(Pt 5):760-763.

2. McCoy AJ, Grosse-Kunstleve RW, Storoni LC, & Read RJ (2005) Likelihood-enhanced fast translation functions. *Acta Crystallogr. D Biol. Crystallogr.* 61(Pt 4):458-464.

3. Perrakis A, Morris R, & Lamzin VS (1999) Automated protein model building combined with iterative structure refinement. *Nat. Struct. Biol.* 6(5):458-463.

4. Adams PD*, et al.* (2010) PHENIX: a comprehensive Python-based system for macromolecular structure solution. *Acta crystallographica. Section D, Biological crystallography* 66(Pt 2):213-221.

5. Emsley P & Cowtan K (2004) Coot: model-building tools for molecular graphics. *Acta Crystallogr. D Biol. Crystallogr.* 60(Pt 12 Pt 1):2126-2132.

6. Coskuner O & Wise-Scira O (2013) Structures and free energy landscapes of the A53T mutant-type alpha-synuclein protein and impact of A53T mutation on the structures of the wild-type alpha-synuclein protein with dynamics. *ACS Chem Neurosci* 4(7):1101-1113.

7. Fang L*, et al.* (2014) Rational design, preparation, and characterization of a therapeutic enzyme mutant with improved stability and function for cocaine detoxification. *ACS Chem Biol* 9(8):1764-1772.

8. Khrenova MG, Nemukhin AV, & Domratcheva T (2015) Theoretical Characterization of the Flavin-Based Fluorescent Protein iLOV and its Q489K Mutant. *J Phys Chem B* 119(16):5176-5183.

9. Lee S, Bhattacharya S, Tate CG, Grisshammer R, & Vaidehi N (2015) Structural dynamics and thermostabilization of neurotensin receptor 1. *J Phys Chem B* 119(15):4917-4928.

10. Zhang J, Wang F, & Zhang Y (2015) Molecular dynamics studies on the NMR structures of rabbit prion protein wild type and mutants: surface electrostatic charge distributions. *J Biomol Struct Dyn* 33(6):1326-1335.

11. Beglov D, Roux, B (1994) Finite Representation of an Infinite Bulk System - Solvent Boundary Potential for Computer-Simulations. *J. Chem. Phys.* 100:9050-9063.

12. Jorgensen WL, Chandrasekhar, J, Madura, JD, Impey, R W, & Klein, ML (1983) Comparison of simple potential functions for simulating liquid water. *J Chem Phys* 79:926-935.

13. Darden T, York, D & Pedersen, L (1993) Particle mesh Ewald: An N [center-dot] log(N) method for Ewald sums in large systems. *J Chem Phys* 98:10089-10092.

14. Frenkel DS, B (2002) *Understanding Molecular Simulation: From Algorithms to Applications* (Academic Press, San Diego, CA).

15. Lossos A*, et al.* (1998) Adult polyglucosan body disease in Ashkenazi Jewish patients carrying the Tyr329Ser mutation in the glycogen-branching enzyme gene. *Ann Neurol* 44(6):867-872.

16. Corpet F (1988) Multiple sequence alignment with hierarchical clustering. *Nucleic Acids Res* 16(22):10881-10890.

17. Robert X & Gouet P (2014) Deciphering key features in protein structures with the new ENDscript server. *Nucleic Acids Res* 42(Web Server issue):W320-324.

18. Bao Y, Kishnani P, Wu JY, & Chen YT (1996) Hepatic and neuromuscular forms of glycogen storage disease type IV caused by mutations in the same glycogen-branching enzyme gene. *J Clin Invest* 97(4):941-948.

19. Burrow TA*, et al.* (2006) Non-lethal congenital hypotonia due to glycogen storage disease type IV. *American journal of medical genetics. Part A* 140(8):878-882.

20. Mochel F*, et al.* (2012) Adult polyglucosan body disease: Natural History and Key Magnetic Resonance Imaging Findings. *Annals of neurology* 72(3):433-441.

21. Bruno C*, et al.* (2004) Clinical and genetic heterogeneity of branching enzyme deficiency (glycogenosis type IV). *Neurology* 63(6):1053-1058.

22. Ravenscroft G*, et al.* (2013) Whole exome sequencing in foetal akinesia expands the genotype-phenotype spectrum of GBE1 glycogen storage disease mutations. *Neuromuscular disorders : NMD* 23(2):165-169.

23. Dainese L*, et al.* (2013) Abnormal glycogen in astrocytes is sufficient to cause adult polyglucosan body disease. *Gene* 515(2):376-379.

24. Ubogu EE*, et al.* (2005) Adult polyglucosan body disease: a case report of a manifesting heterozygote. *Muscle & nerve* 32(5):675-681.

25. Sagnelli A*, et al.* (2014) Adult polyglucosan body disease in a patient originally diagnosed with Fabry's disease. *Neuromuscular disorders : NMD* 24(3):272-276.

26. Magoulas PL*, et al.* (2012) Diffuse reticuloendothelial system involvement in type IV glycogen storage disease with a novel GBE1 mutation: a case report and review. *Hum Pathol* 43(6):943-951.

27. L'Hermine-Coulomb A*, et al.* (2005) Fetal type IV glycogen storage disease: clinical, enzymatic, and genetic data of a pure muscular form with variable and early antenatal manifestations in the same family. *American journal of medical genetics. Part A* 139A(2):118-122.

28. Li SC*, et al.* (2010) Glycogen storage disease type IV: novel mutations and molecular characterization of a heterogeneous disorder. *Journal of inherited metabolic disease* 33 Suppl 3:S83-90.

29. Ziemssen F*, et al.* (2000) Novel missense mutations in the glycogen-branching enzyme gene in adult polyglucosan body disease. *Annals of neurology* 47(4):536-540.

30. Sindern E*, et al.* (2003) Adult polyglucosan body disease: a postmortem correlation study. *Neurology* 61(2):263-265.

31. Billot S*, et al.* (2013) Acute but transient neurological deterioration revealing adult polyglucosan body disease. *Journal of the neurological sciences* 324(1-2):179-182.

32. Paradas C*, et al.* (2014) Branching enzyme deficiency: expanding the clinical spectrum. *JAMA neurology* 71(1):41-47.

33. Massa R*, et al.* (2008) Adult polyglucosan body disease: proton magnetic resonance spectroscopy of the brain and novel mutation in the GBE1 gene. *Muscle & nerve* 37(4):530-536.

34. Sampaolo S*, et al.* (2014) A novel GBE1 mutation and features of polyglucosan bodies autophagy in Adult Polyglucosan Body Disease. *Neuromuscul Disord*.
